# Ectopic expression of PTTG1/securin promotes tumorigenesis in human embryonic kidney cells

**DOI:** 10.1186/1476-4598-4-3

**Published:** 2005-01-13

**Authors:** Tariq Hamid, Mohammed T Malik, Sham S Kakar

**Affiliations:** 1 Department of Medicine, University of Louisville, Louisville, KY 40202, USA; 2 James Graham Brown Cancer Center, University of Louisville, KY 40202, USA; 3 Department of Biochemistry and Molecular Biology, University of Louisville, Louisville, KY 40202, USA

## Abstract

**Background:**

Pituitary tumor transforming gene1 (PTTG1) is a novel oncogene that is expressed in most tumors. It encodes a protein that is primarily involved in the regulation of sister chromatid separation during cell division. The oncogenic potential of PTTG1 has been well characterized in the mouse, particularly mouse fibroblast (NIH3T3) cells, in which it induces cell proliferation, promotes tumor formation and angiogenesis. Human tumorigenesis is a complex and a multistep process often requiring concordant expression of a number of genes. Also due to differences between rodent and human cell biology it is difficult to extrapolate results from mouse models to humans. To determine if PTTG1 functions similarly as an oncogene in humans, we have characterized its effects on human embryonic kidney (HEK293) cells.

**Results:**

We report that introduction of human PTTG1 into HEK293 cells through transfection with PTTG1 cDNA resulted in increased cell proliferation, anchorage-independent growth in soft agar, and formation of tumors after subcutaneous injection of nu/nu mice. Pathologic analysis revealed that these tumors were poorly differentiated. Both analysis of HEK293 cells transiently transfected with PTTG1 cDNA and analysis of tumors developed on injection of HEK293 cells that had been stably transfected with PTTG1 cDNA indicated significantly higher levels of secretion and expression of bFGF, VEGF and IL-8 compared to HEK293 cells transfected with pcDNA3.1 vector or uninvolved tissues collected from the mice. Mutation of the proline-rich motifs at the C-terminal of PTTG1 abolished its oncogenic properties. Mice injected with this mutated PTTG1 either did not form tumors or formed very small tumors. Taken together our results suggest that PTTG1 is a human oncogene that possesses the ability to promote tumorigenesis in human cells at least in part through the regulation of expression or secretion of bFGF, VEGF and IL-8.

**Conclusions:**

Our results demonstrate that PTTG1 is a potent human oncogene and has the ability to induce cellular transformation of human cells. Overexpression of PTTG1 in HEK293 cells leads to an increase in the secretion and expression of bFGF, VEGF and IL-8. Mutation of C-terminal proline-rich motifs abrogates the oncogenic function of PTTG1. To our knowledge, this is the first study demonstrating the importance of PTTG1 in human tumorigenesis.

## Background

Pituitary tumor transforming gene 1 (PTTG1), a recently characterized oncogene, was initially identified on analysis of a rat pituitary tumor [1]; subsequently, a human homologue of PTTG1 was cloned by us and others [2-4]. Three members (PTTG1, PTTG2 and PTTG3) of the PTTG family, which exhibit differential expression in normal and tumor cells have been reported [5], although only PTTG1 has been studied in detail. PTTG1 is located on chromosome 5q35.1 [6], a locus associated with recurrent lung cancer and myelogenous leukemias [7]. Moreover, it has been shown to be expressed highly in various tumors, and cell lines derived from such tumors, including tumors of the pituitary, thyroid, colon, ovary, testicles, and breast [8-12]. In normal tissues, its expression is low or undetectable except in testis [4,1]. Recent studies have indicated that elevated expression of PTTG1 in some tumors may serve as a prognostic marker for tumor invasiveness and metastasis [13]. A clue to its function was gained from its structural similarity with the yeast securin, which led to its identification as a human securin [14] and suggested that it may play a role in regulation of sister chromatid separation. It appears, however, to have multiple effects in cells with enhanced expression being associated with an increase in the expression of the c-myc oncogene [15], an increase in the expression of p53 [16,17], an increase in the secretion and expression of basic growth factor (bFGF) [18] and an increase in the secretion and expression of vascular endothelial growth factor [VEGF) [18,19].

To date, the evidence for the oncogenic function of PTTG1 has been obtained by overexpression of PTTG1 in mouse fibroblast cells (NIH3T3) followed by assessment of its ability to induce cellular transformation in vitro (colony formation in soft agar) and tumor formation in nude mice [2,4]. Due to the biological differences between human and rodent cells, however, care must be taken in extrapolating results obtained using rodent cells to human cells. There are now a number of examples in which it has been demonstrated that although overexpression of an oncogene can induce transformation of primary rodent cells [20], it fails to induce transformation of the same cell type derived from humans. Usually this failure is attributable to the requirement for co-expression of another gene or oncogenic cooperation of another gene [21-26].

Similarly, much of the evidence concerning the mechanisms by which PTTG1 may affect the phenotype of the cell has been obtained using transfected NIH3T3 cells. It is known that the secretion of growth factors and cytokines by tumor cells, and the cells that infiltrate and surround the tumor mass play an essential role in the regulation of tumor growth and metastasis [27]. Both bFGF and VEGF have been implicated in tumorigenesis and the expression and secretion of these molecules has been demonstrated on transfection of NIH3T3 cells with PTTG1 cDNA [18,19], but this has not been confirmed on transfection of human cells. The effects of expression of PTTG1 on another cytokine that is known to play a key role in tumorigenesis, interleukin-8, (IL-8), have not yet been analyzed.

The purposes of this study were, therefore, three-fold. Firstly, to determine whether PTTG1 can induce cellular transformation of normal human cells; secondly, to determine if PTTG1 is sufficient in itself to induce transformation; and, thirdly, to characterize the changes in secretion and expression of key metastatic, angiogenic and chemokine factors (bFGF, VEGF and IL-8) associated with PTTG1-mediated transformation in human cells. For these studies, we selected the human embryonic kidney (HEK293) cell line as our model. The transformation of these cells by human adenovirus type 5 prevents their senescence [28]. These cells have been reported to have a moderate tumorigenic potential [29] and have been used as a cellular model for normal human cells to study the oncogenic potential of a number of genes [29-31]. Mice xenografted with these cells do not develop tumors even after three months of injection [31].

## Results

### Generation of HEK293 cells stably expressing PTTG1 and mPTTG1

**Figure 1:**
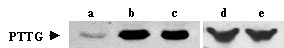
Western blot analysis of HEK293 cells transfected with pcDNA3.1, pcDNA3.1-PTTG1 or pcDNA3.1-mPTTG1. a: HEKpcDNA3.1, b: HEKPTTG1-1, c: HEKPTTG1-3, d: HEKmPTTG1-2, and, e: HEKmPTTG1-4 cells.

HEK293 cells were transfected with pcDNA3.1-PTTG1, pcDNA3.1-mPTTG1 or pcDNA3.1 vector. After G418 selection, 10 clones from each of pcDNA3.1, pcDNA3.1-PTTG1 or pcDNA3.1-mPTTG1 transfected cells were picked, cultured and expanded. The PTTG1 protein expression of these transfectants was detected by western blot analysis using PTTG1 antiserum. Two representative clones from PTTG1 transfected (named HEKPTTG1-1 and HEKPTTG1-3) and mPTTG1 transfected (named HEKmPTTG1-2 and HEKmPTTG1-4), and one clone from pcDNA3.1 vector (named HEKpcDNA3.1) was selected for further studies. Selection of clones was based on the level of expression of PTTG1 protein. Fig. 1 shows the protein expression of these clones. Transfection of cells with the pcDNA3.1 vector resulted in expression of very low level of PTTG1 protein. The clones of the pcDNA3.1-PTTG1 and pcDNA3.1-mPTTG1 transfected cells that exhibited approximately equivalent levels of expression of PTTG1 and mPTTG1 proteins were processed to establish stable cell lines.

### Stable transfection of PTTG1 induces cell proliferation and transformation of HEK293 cells overexpressing PTTG1

**Figure 2:**
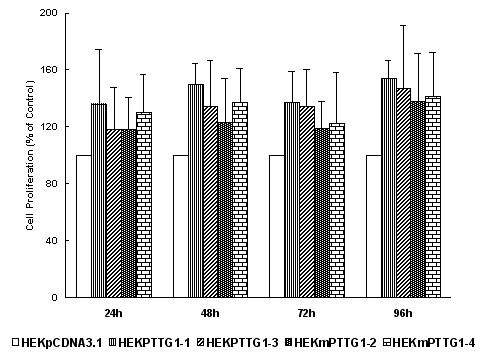
Cell proliferation of HEK293 cells stably transfected with pcDNA3.1, PTTG1 or m-PTTG1. The 5 × 10^3 ^cells were plated/well. The results are expressed as % of control (HEK293 cells stably transfected with pcDNA3.1 control vector). Error bars represent ± SEM (n = 4) of three independent experiments.

Previously, we have shown that over expression of PTTG1 in mouse fibroblast NIH3T3 cells results in an increase in cell proliferation [4]. To determine if over expression of PTTG1 in HEK293 cells produces similar effects, we estimated the proliferation at 24, 48, 72 and 96 hours after plating of stably transfected HEK293 cells expressing high levels of PTTG1 or mPTTG1 protein. Both clones of PTTG1-transfected cells (HEKPTTG1-1 and HEKPTTG1-3) exhibited significantly greater proliferation than the cells transfected with vector only at all time points tested, and the time course of proliferation was very similar in both clones, increasing by 20–30% after 24 hours, 30–40% after 48 hours. This level of increase in cell proliferation was retained at least up to 96 hours (Fig. 2). Somewhat surprisingly, the proliferation of the cells expressing the mutated PTTG1 was equivalent to that of the cells expressing the wild-type PTTG1 and was significantly higher than that of the cells transfected with vector only (Fig. 2). These experiments indicate that over expression of PTTG1 does induce a significant proliferative effect in HEK293 cells; however, at least under the conditions used, mutation of the proline-rich motifs of PTTG1 does not affect this response.

### Overexpression of PTTG1 induces cellular transformation

**Figure 3:**
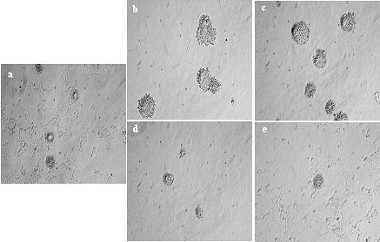
Colony formation of HEK293 cells stably transfected with pcDNA3.1, pcDNA3.1-PTTG1 or pcDNA3.1-m-PTTG1 a: HEKpcDNA3.1, b: HEKPTTG1-1, c: HEKPTTG1-3, d: HEKmPTTG1-2 and e: HEKmPTTG1-4. The data shown is representative of three independent experiments.

As anchorage-independent growth is considered to be an in-vitro test for angiogenesis we assayed the effects of transfection with PTTG1 on the ability of the HEK293 cells to form colonies in soft agar. As shown in Fig. 3, over expression of PTTG1 in HEK293 cells resulted in a higher incidence of colony formation than that observed on transfection with the vector only. The cells transfected with vector only formed few colonies and these were of small size during 14 days of culture, whereas both the cell lines expressing wild type PTTG1 formed a significantly higher number of colonies, which were of a large size. The incidence of colony formation was 2% for HEKpcDNA3.1 cell line but was 19% for the HEKPTTG1-1 cell line and 30% for the HEKPTTG1-3 cell line. In this case, mutation of the proline-rich motifs of PTTG1 resulted in a significant reduction in the number of colonies formed with the incidence of colony formation for the HEKmPTTG1-2 and HEKmPTTG1-4 cell lines being similar to the vector-only transfected cells (HEKpcDNA3.1). These results suggest that over expression of PTTG1 in HEK293 cells induces cellular transformation, and mutation of proline-rich motifs does not effect the cell proliferation but abrogates the cellular transformation ability of PTTG1.

### PTTG1 induces tumor formation in nude mice injected with HEK293 cells stably expressing PTTG1 protein

**Figure 4:**
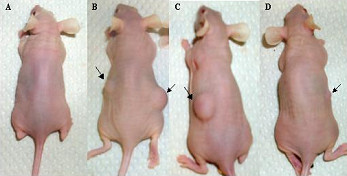
Tumor development in nu/nu mice on injection of HEK293 cells stably transfected with pcDNA3.1, pcDNA3.1PTTG or pcDNA3.1mPTTG1 cells. Each mouse was injected with 1 × 10^6 ^cells. After 6 weeks of injection, mice were photographed and sacrificed, tumors and other tissues were collected and tumor volume was measured a: Mouse injected with HEKpcDNA3.1 cells, b: mouse injected with HEKPTTG1-1 cells, c: mouse injected with HEKPTTG1-3 cells and d: mouse injected with HEKmPTTG1-2. Arrows indicate the tumors.

**Figure 5:**
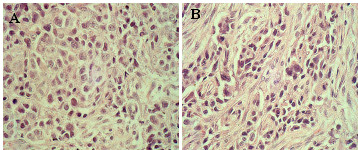
Histolopathological analysis of the tumors excised from animals injected with HEK293 cells expressing PTTG1 or m-PTTG1. Tumors were fixed, sectioned and stained for H & E. A: Tumor from animal injected with PTTG1-1, B: Tumor from animal injected with HEKPTTG1-3.

To determine whether PTTG1 promotes tumor formation in nude mice, we subcutaneously injected nude mice with HEK293 cells expressing PTTG1 or mPTTG1. Three out of four mice injected with the HEKPTTG1-1 or HEKPTTG1-3 cell lines developed large size tumors within four weeks of injection (Fig. 4). Pathologic analysis of the tumors revealed that they were poorly differentiated (Fig. 5). Mice injected with the HEKmPTTG1-2 cell line did develop tumors, but the tumors were of a small size. None of the mice injected with the other cell line-expressing mutant PTTG1 (HEKmPTTG1-4) or the vector-only cell line (HEKpcDNA3.1) developed tumors within the time frame of this experiment (Fig. 4). The tumor volumes, measured at the end of experiment (six weeks after injection of cells), were 150–1320 mm^3 ^for HEKPTTG1-1, 72–1404 mm^3 ^for HEKPTTG1-3 and 8.8–12.6 mm^3 ^for HEKmPTTG1-2 (Table 1). These results clearly demonstrate that PTTG1 gene is a potent oncogene. Moreover, they demonstrate that PTTG1 possesses the ability to enhance the tumorigenic potential of immortal human cells and that it does not require the ectopic co-expression of other oncogene(s) to achieve its tumorigenic function.

**Table 1:** Tumor formation induced by PTTG1 expressing HEK293 cells in nude mice

**Stable Cells**	**Animals with tumor**	**Tumor Volume**
**HEKpcDNA3.1**	0/4	NA
**HEKPTTG1-1**	3/4	150–1320 mm^3^
**HEKPTTG1-3**	3/4	72–1404 mm^3^
**HEKmPTTG1-4**	2/4	8.8–12.6 mm^3^
**HEKmPTTG1-2**	0/4	NA

### PTTG1 stimulates expression and secretion of bFGF, VEGF and IL-8

**Figure 6:**
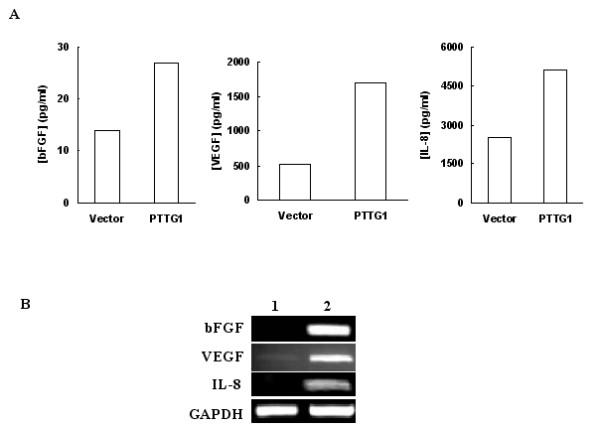
Introduction of PTTG in HEK293 cells induces secretion and expression of bFGF, VEGF and IL-8. HEK293 cells were transiently transfected with pcDNA3.1 or pcDNA3.1-PTTG1 cDNA. The culture medium was collected and lyophilized. bFGF, VEGF and IL-8 secreted in culture medium were measured by ELISA. A: Amount of bFGF, VEGF and IL-8 in culture medium. Vector: cells transfected with pcDNA3.1 vector DNA; PTTG1: Cells transfected with pcDNA3.1-PTTG1 cDNA. B: Expression of bFGF, VEGF and IL-8 mRNA in cells. Lane 1: pcDNA3.1 transfected cells and Lane 2: pcDNA3.1-PTTG1 transfected cells. GAPDH was used as an internal control. The data are representative of two independent experiments.

**Figure 7:**
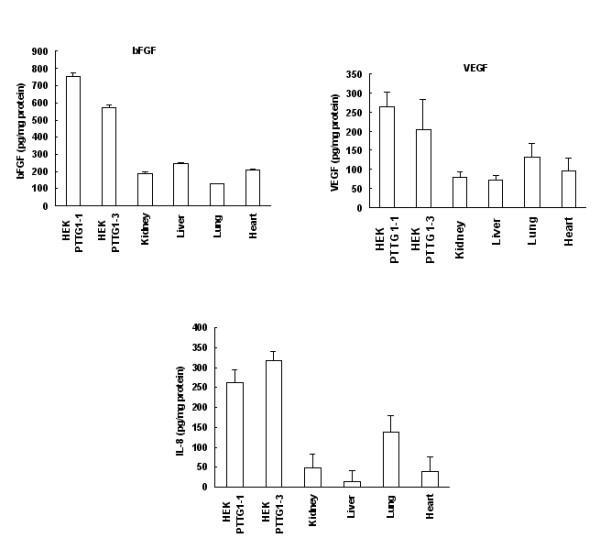
Analysis of bFGF, VEGF and IL-8 expression in tumors and other tissues. Tumors and other tissues were excised from the animals injected with HEK293 cells stably transfected with PTTG1 (clone 1 and clone3) and homogenized. bFGF, VEGF and IL-8 in the homogenates were analyzed by ELISA. Each analysis was performed in triplicate tissue and was normalized to mg of protein. Error bars represent ± SEM of three independent experiments.

**Figure 8:**
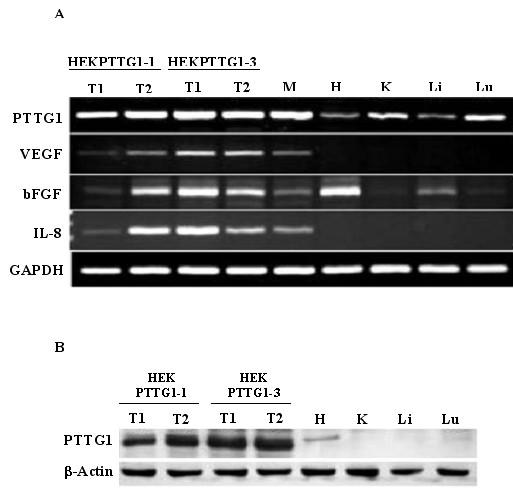
Analysis of expression of PTTG bFGF, VEGF and IL-8 mRNAs from tumors and other tissues collected from mice subcutaneously injected with HEK293 cells stably expressing PTTG1. A: RT-PCR analysis and, B: Western blot analysis. T1: tumor 1, T2: tumor 2, M: HEKmPTTG1-2, H: heart, K: Kidney, Li: liver, Lu: lung. GAPDH and β-actin were used as control to examine equal loading. The gels are representative of two independent experiments.

Local invasive growth is a key feature of primary malignant tumors. A correlation between the levels of expression of PTTG1 with increased tumor invasiveness and with the degree of malignancy has been demonstrated in pituitary and colorectal tumors [9,35]. The specific mechanisms by which PTTG1 facilitates the invasive behaviors of tumor cells remain obscure, however. Recently, Ishikawa et al [18] and McCabe et al [19] have shown that transfection of NIH3T3 cells with PTTG1 cDNA results in an increase in secretion and expression of both bFGF and VEGF. A direct correlation between high IL-8 expression and tumor metastases has been shown in a number of cancers [36-38], and IL-8 also has been reported to possess mitogenic [39] and angiogenic effects [40]. We therefore measured the levels of bFGF, VEGF and IL-8 in HEK293 cells transiently transfected with pcDNA3.1 or pcDNA3.1-PTTG1 cDNA and in tumors developed on injection of nude mice with HEK293 cells that constitutively express PTTG1. As shown in Fig. 6A, the levels of bFGF, VEGF and IL-8 were comparatively higher in conditioned medium of cells transfected with pcDNA3.1-PTTG1 cDNA than from cells transfected with pcDNA3.1 vector only. Cells transfected with pcDNA3.1-PTTG1 showed a 2-fold increase in bFGF, a 3.5-fold increase in VEGF and a 2-fold increase in IL-8 levels compared to cells transfected with pcDNA3.1 vector only. Measurement of the mRNA levels of these factors by RT/PCR showed significantly higher levels in cells transfected with pcDNA3.1-PTTG1 cDNA compared to cells transfected with pcDNA3.1 vector (Fig. 6B). To determine, if over expression of PTTG1 results in increase in levels of bFGF, VEGF and IL-8 in vivo, we measured the levels of bFGF, VEGF and IL-8 proteins in lysates from tumors developed on injection of nude mice with HEK293 cells stably transfected with PTTG1. As shown in Fig. 7, the levels of bFGF, VEGF and IL-8 were significantly higher in three out of four tumors compared to normal tissues (kidney, liver, lung and heart) collected from the same animals. Since the size of the tumors that developed on injection of cells expressing mutated PTTG1 (HEKmPTTG1-2) were small, we were unable to analyze the bFGF, VEGF and IL-8 levels in these tumors. Measurement of mRNA for bFGF, VEGF and IL-8 revealed significantly higher levels of expression in tumors compared to normal tissues and tumors developed on injection of HEKmPTTG1-2 cells. As expected, levels of PTTG and mPTTG were higher in tumors as compared to normal tissues (Fig. 8). bFGF levels were found to be comparatively higher in heart which is consistent with other investigators [41] Taken together our results suggest that over expression of PTTG1 in HEK293 cells results in an increase in secretion and expression of bFGF, VEGF and IL-8 in vitro and in vivo, suggesting that increase in secretion and expression of bFGF, VEGF and IL-8 by PTTG1 may be one of the mechanisms by which PTTG1 achieves its oncogenic function and increases tumor angiogenesis.

## Discussion

Oncogenic function of PTTG1 was established by its overexpression in mouse fibroblast cell line (NIH 3T3) followed by assessment of its ability to induce cellular transformation and tumor formation in nude mice [2,4]. However, the differences in biology between the rodent cells and human cells have brought the validity of this model into question. There are a number of instances in which an oncogene has been shown to induce transformation in rodent cells but has failed to induce transformation of same types of cells obtained from humans. To test the ability of PTTG1 to induce transformation in human cells, we selected the human embryonic kidney-293 (HEK293) cell line as our model. Our data clearly demonstrate that over expression of PTTG1 in HEK293 results in an increase in cell proliferation induces cellular transformation in-vitro (increase in anchorage-independent growth) and promotes tumor formation in nude mice. Cells transfected with pcDNA3.1 vector did not form colonies in soft agar or develop into tumors on implantation in nude mice, confirming that HEK293 cells do not possess a tumorigenic phenotype. Furthermore our data suggest that overexpression of PTTG1 in these cells results in a greater propensity for tumor development, a shortened latency period and an enhanced growth rate compared with pcDNA3.1-transfected control HEK293 cells.

A second issue that we were able to address using the HEK293 cell model was the question of the ability of PTTG1 to induce transformation of normal human cells, i.e., whether it acts alone or in cooperation with another oncogene to achieve its tumorigenic function. It has been reported that a single oncogene may not be sufficient for induction of transformation but requires co-expression, or oncogenic cooperation, of another oncogene(s) to induce tumorigenesis in normal primary human cells [20,21,24,25,42,43]. Our results clearly show that PTTG possess the ability to induce cellular transformation in vitro and promotes tumor formation in nude mice. Since, HEK293 cells are transformed with adenovirus type 5 thus making them different from the normal primary cells, therefore it remains unclear if PTTG is sufficient by itself to initiate the tumorigenesis of primary human cells. However, the data clearly demonstrate that PTTG1 overexpression in these cells accelerates their tumorigenic capacity in comparison to that of unmodified cells.

PTTG1 contains several-proline rich motifs (PXXP); two of these that are located in the C-terminal domain have been reported to be potential binding sites for SH3-domians [44]. In our study we confirm that mutation of these C-terminal proline-rich motifs abrogates the tumorigenicity of PTTG1 in human cells. Such loss of tumorigenicity on mutation of PTTG1 could be due to a loss of expression. Our western blot analysis of the stable cell lines (HEKmPTTG1-2 and HEKmPTTG1-4) that constitutively express mutated PTTG1 protein showed high levels of expression of mPTTG1 protein (Fig. 1), suggesting that the loss of tumorigenic function of mPTTG1 protein is not due to loss of expression but due to the loss of its ability to induce cellular transformation. These results are consistent with other investigators for rodent cells [2] and confirm the importance of C-terminal proline-rich motifs to mediate the oncogenic function of PTTG1.

The molecular mechanisms by which PTTG1 achieves its tumorigenic function remain unclear. PTTG1 has been reported to induce expression of the c-myc oncogene [15], bFGF [18] and VEGF [19]. bFGF is a broad spectrum and pleiotropic mitogen for growth and differentiation affecting various mammalian cells and organ systems and a large number of cells lines [45,46]; besides stimulating wound healing, tissue repair and hematopoiesis [47], bFGF induces cell migration and proliferation [48] and acts as an agiogenic factor that induces migration, proliferation and differentiation of endothelial cells [49]. In addition, it has been reported to modulate the invasion of tumor cells through surrounding tissue to form new capillary cord structures by regulating the activities of extracellular molecules including collagenase, proteinases and integrins [49]. Regulation of secretion and expression of bFGF by PTTG1 in NIH 3T3 cells has been shown [18]. Consistent with these reports, our results demonstrate a significant increase in secretion and expression of bFGF in HEK293 cells on transient transfection with PTTG1 cDNA (Fig. 6) as well as in tumors developed by injection of stable cell lines that constitutively express PTTG1 both at protein and mRNAs levels (Figs. 6, 7, 8).

VEGF is a potent stimulant of the vascularization of tumors and is one of the most specific markers of tumor vasculature observed to date [50,51]. VEGF is a multifunctional cytokine acting as a potent permeability agent, an endothelial cell chemotactic agent, an endothelial cell survival factor and an endothelial cell proliferation factor [52]. The expression and secretion of VEGF has been shown to be a crucial rate-limiting step during tumor progression [53]. Our results demonstrate a significant increase in secretion and expression of VEGF in HEK293 cells on transfection with PTTG1 and also from tumors excised from animals injected with HEK293 cells that stably express PTTG1 (Figs. 6, 7, 8).

A direct correlation between high IL-8 expression and metastases in melanoma [36], ovarian cancer [37], prostrate cancer [38] and pancreatic cancer [51] has been reported. To determine if overexpression of PTTG1 induces change in secretion and expression of IL-8, we measured its levels in HEK293 cells on transfection with PTTG1 cDNA and in tumors developed on injection of HEK293 cells transfected with PTTG1. Our results demonstrate for the first time that overexpression of PTTG1 induces IL-8 expression in vitro and also in tumors in vivo (Figs. 6, 7, 8).

## Methods

### Generation of cell lines constitutively expressing PTTG1

The human embryonic kidney cell line (HEK293), which had been transfected by exposing these cells to sheared fragments of adenovirus type 5 DNA [28] was purchased from ATCC (American Type Culture Collection; Rockville, MD) and cultured according to the instructions provided. The cells were transfected with pcDNA3.1 vector, pcDNA3.1-PTTG1 or pcDNA3.1-mPTTG1 to generate stable clones that constitutively express human wild-type PTTG1 or mutated PTTG1 (mPTTG1) protein as described previously [4]. The mPTTG1, which carries a double amino acid change within the SH3 binding domain of PTTG1 (P^163 ^to A^163^, P^170 ^to A^170 ^and P^172 ^to A^172^, and P^173 ^L^173^), was generated by site-directed mutagenesis using the Quick-change mutagenesis kit (Stratagene, La Jolla, CA) according to the manufacturer's instructions. Mutation of these amino acids has been reported to abrogate the tumorigenic function of PTTG1 and to block the secretion and expression of bFGF in mouse NIH3T3 cells [2]. The primers used for this site-directed mutagenesis were 5'-GATGCTCTCCGCACTCTGGGAATCCAATCTG-3' and 5'-TTCACAAGTTGAGGGGCGCCCAGCTGAAACAG-3'. The transfected cells were then selected in neomycin G418 (500 μg/ml) and the clones that expressed high levels of PTTG1 protein or mPTTG1 protein were selected. One clone from pcDNA3.1 transfected cells (HEKpcDNA3.1) two clones from pcDNA3.1-PTTG1 transfected cells (HEKPTTG1-1 and HEKPTTG1-3) and two clones from pcDNA3.1-mPTTG1 transfected cells (HEKmPTTG1-2 and HEKmPTTG1-4) were propagated into cell lines.

### Cell proliferation assay

Cell proliferation was assayed using the CellTiter 96 non-radioactive cell proliferation assay kit (Promega, Madison, WI) according to the manufacturer's instructions and as described previously [4]. Briefly, cells growing in log phase were trypsinized and seeded in 96-well plates (5,000 cells/well in a final volume of 100 μl) in replicates of 4 and incubated at 37°C in 5% CO_2 _and 95% air. After incubation for 24 h, 48 h, 72 h or 96 h, 20 μl of dye solution from the kit was added to each well and incubated at 37°C for an additional 2 h. The quantity of formazon product was measured by its absorbance at 490 nm using a 96-well plate reader (Molecular Devices, Sunnyvale, CA). Each experiment was repeated at least three times.

### Soft agar colony formation (anchorage-independent cell growth) assay

Anchorage-independent cell growth was determined by analyzing the formation of colonies in soft agar. Cells (10^4^) from each cell line were suspended in 0.3% agar in DMEM containing 10% fetal bovine serum and plated on solidified agar (0.7%) in 35 mm dishes. After 14 days of culture, colonies formed were counted and photographed as described previously [4].

### In vivo tumor growth assay

Cells growing in log phase were harvested by trypsinization and washed twice with PBS. The cells were resuspended in PBS to a final concentration of 5 × 10^6^/ml. The cells (1 × 10^6 ^cells in 200 μl PBS/site) were injected subcutaneously (s.c.) into both flanks of 5- to 6-week old female nu/nu mice (4 mice/group) (Charles River Laboratory, Wilmington, MA). All procedures were carried out following the protocol approved by The University of Louisville Institutional Animal Care and Use Committee. Four weeks after injection, the mice were scarified, and the tumors and other tissues harvested. The skin and connective tissues were dissected from the tumors, and the tumor volume was calculated from measurements of length × width × height. The tissues were divided into two parts, one part being fixed in 10% buffered formalin and the other stored in liquid nitrogen. For histopathologic analysis, 5 μm sections were cut from paraffin-embedded tissues, and mounted on slides. Sections were stained with H&E [52], and processed for histopathologic evaluations.

### Western blot analysis

Cells growing in log phase were lysed in chilled lysis buffer [50 mM Tris-HCl (pH 7.5), 150 mM NaCl, 1% NP-40, 1 mM Na_3_VO_4_, and 1 mM NaF) supplemented with Complete Mini Protease Inhibitor tablets (Roche Molecular Biochemicals, Indianapolis, IN). Equal amounts of protein extracts (40 μg) were resolved on 12% SDS-PAGE gel, and transferred onto a nitrocellulose membrane (Amersham, Piscataway, NJ). Blots were probed with PTTG1 antiserum at a dilution of 1:1,500 as described previously [53]. Immunoreactive proteins were visualized using the Enhanced Chemiluminescent Detection System (Amersham) according to the instructions provided.

### ELISA analysis of bFGF, VEGF and IL-8

The levels of bFGF, VEGF and IL-8 in tissue culture supernatants and tissue homogenates were measured using commercially available ELISA kits from BD Biosciences (Minneapolis, MN). To measure bFGF, VEGF and IL-8 in the culture supernatants, HEK293 cells were transiently transfected with pcDNA3.1 or pcDNA3.1-PTTG1 cDNA using Fugene6 as the transfectant reagent as described previously [54]. After 24 h of transfection, the medium was replaced with serum free DMEM medium. Twenty-four later, the medium was collected and concentrated 5-fold (1.0 ml to 200 μl) using a speedVac system (Savant, Holbrook, NY). To measure bFGF, VEGF and IL-8 in tumor and other tissues, tissues were homogenized in 50 mM Tris (pH 7.4), 0.25% Triton X-100, 5 mM EDTA and 0.1% NP40 supplemented with Complete Mini Protease Inhibitor tablets (Roche Molecular Biochemicals, Indianapolis, IN) using a polytron homogenizer. Homogenates were centrifuged to remove particulate matter and then diluted with the diluent provided in the ELISA kit. The concentration of bFGF, VEGF and IL-8 in a sample was determined by interpolation from a standard curve. All measurements were normalized to protein concentration and performed in triplicate.

### Semiquantitative reverse transcriptase/polymerase chain reaction (RT/PCR)

Total RNA from tumors and other tissues was purified using Trizol reagent (Invitrogen, Carlsbad, CA) following the manufacturer's instructions. The RNA pellets were resuspended in RNase-free water, and the contaminating DNA was removed from the preparations with DNaseI. The yield of total RNA was measured using a spectrophotometer, and the quality was assessed by electrophoresis through a 1% agarose gel. First strand cDNA was synthesized using the iScript™ cDNA synthesis kit (BioRad, Hercules, CA). PCR primers (Table 2) were designed, based on the human PTTG1, bFGF, VEGF and IL-8 cDNA sequences. The PCR conditions for each gene are listed in Table 1. GAPDH amplification was used as an internal control. Ten μl from a total of 50 μl PCR reaction mix was applied to a 2% agarose gel and after electrophoresis; the gel was stained with ethidium bromide to visualize PCR products. The densitometric values for the PCR-amplified products were quantified using BioRad software and normalized against the GAPDH values.

**Table 2:** Primer sequences and PCR conditions for the amplification of PTTG1, bFGF, VEGF, IL-8, m-PTTG1 and GAPDH.

	**Sense Primer**	**Antisense Primer**	**PCR Conditions**
**PTTG**	ATGGCTACTCTGATCTAT	AAAATCTATGTCACAGCAAAC	95°C 5 min, 95°C 30 s, 54°C 30 s, 72°C 30 s. 28 cycles.
**bFGF**	TTCTTCCTGCGCATCCACCC	CTCTTAGCAGACATTGGAAG	95°C 5 min, 95°C 30 s, 56°C 30 s, 72°C 30 s. 26 cycles.
**VEGF**	GAATCATCACGAAGTGGTGA	AACGCGAGTCTGTGTTTTTG	95°C 5 min, 95°C 30 s, 56°C 30 s, 72°C 30 s. 28 cycles.
**IL-8**	ACCACCGGAAGGAACCATCT	GAATTCTCAGCCCTCTTCAA	95°C 5 min, 95°C 30 s, 58°C 30 s, 72°C 30 s. 28 cycles.
**GAPDH**	TGATGACATCAAGAAGGTGGT	TCCTTGGAGGCCATGTGGGCC	95°C 5 min, 95°C 30 s, 54°C 30 s, 72°C 30 s. 26 cycles.
**mPTTG**	GATGCTCTCCGCACTCTGGGAATCCAATCTG	TTCACAAGTTGAGGGGCGCCCAGCTGAAACAG	95°C 5 min, 95°C 30 s, 54°C 30 s, 72°C 30 s. 35 cycles.

## Conclusion

In summary, our results demonstrate that PTTG1 is a potent human oncogene and has the ability to induce cellular transformation of human cells. Overexpression of PTTG1 in HEK293 cells leads to an increase in the secretion and expression of bFGF, VEGF and IL-8. Mutation of C-terminal proline-rich motifs abrogates the oncogenic function of PTTG1. To our knowledge, this is the first study demonstrating the importance of PTTG1 in human tumorigenesis.

## Authors' contributions

TH carried out the in-vivo studies, RT-PCR analysis, western blot analysis and drafted the manuscript. MTM generated stable clones for HEKmPTTG1-2 and 4, RT-PCR and ELISA analysis. SSK participated in study design and coordination and generated stable cell lines for HEKPTTG1-1 and 3. All authors read and approved the final manuscript.
